# Evaluating the learning curve for retroperitoneoscopic adrenalectomy in a high-volume center for laparoscopic adrenal surgery

**DOI:** 10.1007/s00464-016-5284-0

**Published:** 2016-10-17

**Authors:** A. van Uitert, F. C. H. d’Ancona, J. Deinum, H. J. L. M. Timmers, J. F. Langenhuijsen

**Affiliations:** 1 Department of Urology, Radboud University Medical Centre, Geert Grooteplein Zuid 10, 6525 GA Nijmegen, The Netherlands; 2 Department of Internal Medicine, Radboud University Medical Centre, Geert Grooteplein Zuid 10, 6525 GA Nijmegen, The Netherlands

**Keywords:** Adrenal surgery, Laparoscopic surgery, Retroperitoneoscopic surgery, Benign adrenal disease

## Abstract

**Background:**

Laparoscopic adrenalectomy is an effective method for benign adrenal tumor removal. In the literature, both lateral transperitoneal (TLA) and posterior retroperitoneoscopic (RPA) approaches are described. Since 2007, the number of patients increased significantly in our center. Therefore, RPA was introduced in 2011 because of its potential advantages in operating and recovery times. The learning curve of RPA is now evaluated.

**Methods:**

All data of patients undergoing laparoscopic adrenalectomy from 2007 until 2014 were prospectively collected. Patients were eligible for RPA with a tumor <7 cm, with BMI < 35 kg/m^2^, and with low suspicion of malignancy. The learning curve of RPA was measured by operating time. Furthermore, blood loss, preoperative complications and hospital stay were analyzed. Descriptive statistics were performed using SPSS 20.0.

**Results:**

In the study period, 290 patients underwent surgery, of whom 113 underwent RPA. After starting with RPA, operating times decreased significantly (median 100 min in the first 20 patients to 60 min after 40 patients, *p* < 0.05). There was a significant difference in operating times (median 108 vs. 62 min, *p* < 0.05) and hospital stay (median 4 vs. 3 days, *p* < 0.05) in unilateral surgery in favor of RPA, compared to TLA. Also in bilateral surgery, operating times were significantly shorter (median 236 vs. 117 min, *p* < 0.05). In both groups, few major complications occurred.

**Conclusion:**

After the introduction of RPA, a short learning curve was seen for a single surgeon with extensive experience in laparoscopic adrenal surgery. Compared to TLA, RPA has significant advantages in operating times and hospital stay. Therefore, RPA may be the preferred approach for patients with BMI < 35 kg/m^2^ and small benign adrenal tumors (<7 cm).

In 1992, Gagner et al. [1] first described the technique of transperitoneal laparoscopic adrenalectomy. The first retroperitoneoscopic adrenalectomy (RPA) was reported in 1994 [2]. Compared to open adrenal surgery, laparoscopic surgery is associated with less blood loss and shorter hospital stay [3, 4]. Nowadays, it is generally accepted that the laparoscopic approach is the standard approach for small benign adrenal tumors. Also in large benign tumors and pheochromocytomas laparoscopic adrenalectomy is proven to be safe and effective [5]. The feasibility and safety of both transperitoneal and retroperitoneoscopic approaches have been proven; however, there is certain debate about which technique is superior. In a recent meta-analysis published by Chai et al. [6], comparing both techniques, in which eight prospective studies were evaluated, RPA was shown to be slightly superior when comparing amounts of blood loss and duration of hospital stay, although the included studies dealt with few patients. A recent RCT by Barczynski et al. [7] showed excellent results of both techniques in unilateral small tumors, but a statistically significant advantage of RPA with regard to operating times, blood loss, postoperative pain, and recovery after surgery. A large retrospective study by Walz et al. [8] showed excellent results of RPA. Although RPA is widely performed it may have a longer learning curve, because a paucity of anatomical landmarks “en route” exists.

During the implementation of new surgical techniques, two paths of learning curve can be distinguished. In the first phase, in which a completely new technique is being developed, the learning curve is long. In the second phase, in which a newly developed technique is introduced to another clinic with an experienced surgeon, the learning curve is normally much shorter. In our hospital, two experienced laparoscopic urologists have performed a minimum of 50 laparoscopic renal en adrenal operations each per year for over 8 years. One of these urologists has performed RPA from the beginning of 2011 onwards for small benign adrenal tumors. Although the popularity of RPA is increasing, there is limited evidence describing the learning curve. Furthermore, the incidence of small adrenal tumors is increasing, requiring improvement in efficiency and operating times. Therefore, the purpose of this study is to evaluate the learning curve for RPA of an experienced laparoscopic urologist after phase-two introduction and to compare the outcome of both techniques.

## Materials and methods

### Patient selection

For patients undergoing laparoscopic adrenalectomy from February 2007 until December 2014, all standard perioperative data were collected in a prospective database. Since patients were not subjected to investigational actions, no informed consent was obtained. Patient confidentiality was guaranteed according to the Dutch law on personal data protection. Patients underwent laparoscopic adrenal surgery for different indications, including primary aldosteronism, Cushing syndrome, pheochromocytoma, non-functioning adenoma or suspected adrenocortical carcinoma, and metastases. Preoperatively all patients were evaluated with a computed tomography (CT) to evaluate position and size of the adrenal tumor. In case of primary aldosteronism, adrenalectomy was performed based on findings of CT and/or selective adrenal venous sampling to determine unilaterality, if present, of aldosterone hypersecretion, as part of the SPARTACUS trial [9]. Patients with a pheochromocytoma were admitted preoperatively at the endocrinology department for adequate regulation of blood pressure with pharmacological alpha and beta blockade. Because of a dramatic increase in patients referred to the clinic, RPA was introduced in February 2011 for its potential benefits in operating times and hospital stay. From 2011, patients were eligible for RPA with a body mass index (BMI) of <35 kg/m^2^, with a tumor diameter <7 cm, and with low suspicion of malignancy. Otherwise, TLA was performed. Before introducing RPA, both urologists had already completed the learning curve for TLA. One urologist (JFL) was trained intensively by visiting and proctoring of an expert in RPA (prof. M.K. Walz) before performing this technique independently.

### Surgical technique

TLA patients were positioned in the lateral decubitus position. Three to four trocars were used during left adrenalectomy and an extra trocar during right adrenalectomy for liver retraction. In case of bilateral adrenalectomy, the patient was turned to the opposite side in the course of the operation. RPA was performed in prone position. Three trocars were required for both left and right adrenalectomy. No repositioning was required in case of bilateral surgery. The technique used was described in detail by Walz et al. [10]. RPA was performed by a single surgeon and TLA by both.

### Data collection

Preoperative data included demographics, comorbidity, indication for surgery, surgical technique, blood loss, operating times, hospital stay, and postoperative complications. Operating time was calculated by skin-to-skin time. Hospital stay was defined from day of surgery until day of discharge. Long-term complications were assessed by reviewing the outpatient charts. Complications were scored using the Clavien–Dindo scoring system including postoperative complications during 30 days [11].

### Statistical analysis

Statistical analysis was performed using SPSS 20.0 for Windows (SPSS Inc, Chicago, IL, USA). Normality was tested using the Kolmogorov–Smirnov test. In case of normality, continuous outcomes are displayed as means (±standard deviation) and compared using the independent sample *t* test, and in case of skewed distribution, outcomes are displayed as medians (±interquartile range) and compared using nonparametric tests. Linear regression analysis was performed to determine pattern and duration of learning curve.

## Results

In total, 290 laparoscopic adrenalectomies were performed. For baseline characteristics, see Table 1. Both RPA and TLA were frequently performed in 113 and 177 patients, respectively. When comparing baseline patient characteristics of both groups, the only significant difference was seen for BMI (median 27 vs. 26 kg/m^2^, *p* = 0.03). The percentage of pheochromocytomas was comparable in both groups (23.7 vs. 22.1 %), and suspected malignant tumors were operated only by TLA.

**Table 1:** Descriptive statistics of patients undergoing laparoscopic adrenalectomy

*n*	290
Sex (M/F)	141/149
Age (years)^a^	51 (13)
Body mass index (kg/m^2^)^a^	28 (5)
Indication of surgery	
Prim. aldosteronism	141
Pheochromocytoma	67
Cushing syndrome	44
Non-functioning adenoma	18
Metastasis	9
Adrenocortical carcinoma	3
Other	8
Surgical approach	
Transperitoneal laparoscopic	177
Posterior retroperitoneoscopic	113
Side	
Left	155
Right	112
Bilateral	23
Diameter of tumor (cm)^a^	2.8 (2.2)

^a^Mean (±SD)

**Fig. 1:**
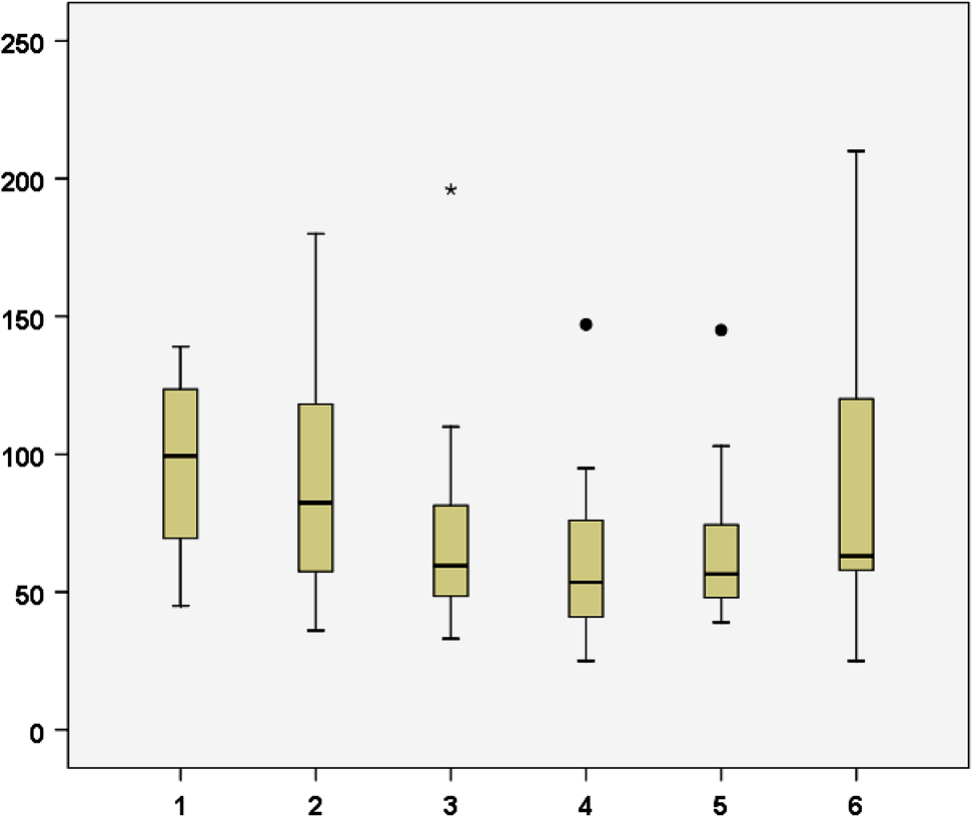
Surgical duration dichomotized per 20 patients. *X-*axis groups of patients (per 20), *Y-*axis operating time (min)

From 2011, there was a clear learning curve for RPA, with a significant decrease in median operating time when comparing the first 20 patients to patients 21–40 and 41–60 (100 min to 83 min to 60 min, *p* < 0.05) (Fig. 1). When performing linear regression analysis, the learning curve showed a quadratic pattern, with a first-grade coefficient of −1.403 (CI −2.229 to −0.577), second-grade coefficient of 0.01 (CI 0.003–0.017), and *R* ^2^ of 0.109. The regression coefficient reaches zero after 70 patients. There was no significant decrease in blood loss, conversion rate, hospital stay, preoperative complications, or postoperative complications.

Two patients required preoperative and postoperative blood transfusions for blood loss, one in both groups. In six patients, a strategic conversion to TLA was performed at the start of the RPA due to limited exposure. Conversion to open surgery was necessary in 10 patients, of whom 8 were in TLA. In total, 33 patients developed a preoperative complication, similar in both groups. In RPA, there was one Clavien IV complication (caval lesion resulting in major blood loss), and in TLA one Clavien IV complication (mesenterial thrombosis) and one Clavien V complication (cecal blowout, resulting in death). There were no other specific complications as a result of positioning or access technique.

When analyzing matched cohorts, by excluding patients who were not eligible for RPA due to an operation date before 2011, BMI ≥ 35 kg/m^2^, tumor size ≥7 cm, or suspected malignancy, there was a significant difference in operating times (90 vs. 62 min, *p* < 0.05) and hospital stay (4 vs. 3 days, *p* = 0.02) in favor of RPA. After completing the learning curve (40 patients), the difference in operating times increased even more (90 vs. 57 min, *p* < 0.05) (Table 2). Both urologists performed TLA in this group and showed similar results in operating times (86 vs. 93 min, *p* = 0.74) and hospital stay (4 vs. 4 days, *p* = 0.57). Also in bilateral surgery, there was a significant difference in blood loss (40 vs. 5 cc, *p* < 0.05) and operating times (236 vs. 117 min, *p* < 0.05) in favor of RPA.

**Table 2:** Matched cohort analysis unilateral TLA versus RPA after 40 patients

	Unilateral TLA (*n* = 38)	Unilateral RPA (*n* = 64)
Blood loss (cc)^a^	10 (45)	5 (5)^b^
Operating time (min)^a^	90 (39)	57 (26)^b^
Hospital stay (days)^a^	4 (2)	3 (1)^b^
Post-op complications		
Clavien I–II	3	5
Clavien III	0	0
Clavien IV–V	0	1

^a^Median (IQR)

^b^ *p* < 0.05

In unilateral RPA, there was a difference in operating times between male and female patients (73 vs. 54 min, *p* = 0.005), indicating surgery to be faster in female patients. In TLA, patients with a pheochromocytoma had more blood loss (median 100 vs. 20 cc, *p* < 0.000), longer operating times (median 117 vs. 100 min, *p* = 0.06), and longer duration of admission (median 5 vs. 4 days, *p* < 0.000), compared to other indications. Also in RPA, these patients had more blood loss (median 15 vs. 5 cc, *p* < 0.000), duration of surgery (median 69 vs. 59 min, *p* < 0.09), and longer duration of admission (median 4 vs. 3 days, *p* < 0.000). Patients with a pheochromocytoma had most conversions to open surgery (6 cases), both in RPA (2 cases) and TLA (4 cases).

## Discussion

In this study, 290 laparoscopic adrenalectomies performed over a period of 8 years are described. Operating times of RPA decreased significantly in time, completely stabilizing after 70 procedures, which is comparable to results in the literature [12–14]. To our knowledge, this study is one of the largest cohorts in which the learning curve of RPA is evaluated. When comparing operating times, the learning curve in this study shows a similar pattern to the introduction phase in the study by Barczynski et al. (110 min directly after the introduction, declining to 75 min after 20 patients, and 65 min after 40 patients). In comparison, operating times that were described for the invention phase were much longer (170 min directly after the introduction, reducing to 110 min after 20 patients, and 95 min after 40 patients) [15]. Lin et al. [14] showed a similar learning curve of 60 patients, when looking at blood loss and operating times, for lateral retroperitoneoscopic adrenalectomies. Cabalag et al. [12] showed a short learning curve of 10 patients in RPA (110–60 min) after an intensive training course with an expert, also pointing to a short learning curve when properly trained.

Most prospective RCTs that have been published comparing RPA and TLA slightly favor RPA in operating times, summarized by Chai et al. [6]. Blood loss and operating times in this study are comparable to the literature, for both techniques. Furthermore, our data suggest an advantage for RPA when comparing blood loss, operating times and hospital stay, which is similar to a recent RCT published by Barczynski et al. [7]. Although the difference in blood loss is not clinically relevant, the reduction in operating time indeed is. Especially in bilateral surgery, operating times can be greatly reduced. Therefore, RPA may be the preferred approach in patients with a body mass index (BMI) of <35 kg/m^2^, with a tumor diameter <7 cm, and with low suspicion of malignancy. Our preoperative outcomes are consistent with data in the literature describing the preoperative results of an experienced laparoscopic surgeon [16]. In this study, a favorable outcome for female patients is seen with RPA. In our experience, this is due to less adherent Gerota fat which enables freeing the upper pole of the kidney more easily. There was no difference when comparing patients with higher BMI, neither in RPA nor in TLA. In patients where RPA was not deemed feasible after the introduction of the optic trocar, conversion to TLA was a good exit strategy, thus preventing conversion to open surgery. There were no specific complications as a result of positioning or access technique. When a malignant lesion is suspected, TLA should be the technique of choice, due to increased exposure and resulting lower chance of tumor spill.

This study has some limitations. First of all, since not all patients were eligible for RPA, there is a selection bias. However, all baseline characteristics were comparable except for BMI and this parameter did not seem to influence outcomes in univariate analysis. Also, in the matched cohort series the differences in outcome were consistent. Secondly, only one urologist performed RPA, making a comparison between both techniques more difficult. However, both urologists showed similar results when comparing outcomes of surgery in TLA, pointing to a similar level of skill. The two urologists performed the surgery in a high-volume center using a dedicated surgical team, making these results hard to extrapolate to general hospitals.

In the future, it is planned to develop a nomogram for an individualized selection of surgical strategy for patients with benign adrenal tumors, using both anatomical and general patient characteristics. This will need validation in future studies.

## Conclusion

In conclusion, operating times of RPA for an experienced laparoscopic urologist stabilized at 70 patients, after intensive training by an expert. Both TLA and RPA were associated with minimal amounts of blood loss, short operating times, and hospital stay. Furthermore, there were few major complications. RPA seems to be superior in patients with small benign adrenal tumors (<7 cm) and BMI < 35 kg/m^2^; however, this needs to be further validated in prospective randomized studies.
